# Exploring work engagement and its associated factors among supervising pharmacists in Japan

**DOI:** 10.1016/j.rcsop.2025.100691

**Published:** 2025-12-05

**Authors:** Yukari Ito, Hiroko Suzuki, Tetsuro Yumoto, Sachiko Ohta, Tomoo Hosoe

**Affiliations:** a School of Pharmacy and Pharmaceutical Sciences, Hoshi University, Japan; b Center for Health Service, Outcomes Research and Development, Japan; c Department of Pharmaceutical and Medical Business Sciences, Nihon Pharmaceutical University, Japan; d Shonan University of Medical Sciences, Japan

**Keywords:** Work engagement, Supervising pharmacists, Occupational stress, JD-R model, Career development, UWES-17, Communities of practice

## Abstract

**Background:**

This study investigated the work engagement of supervising pharmacists and sought to identify factors associated with high work engagement.

Given the growing shortage of healthcare professionals in rapidly aging societies, enhancing work engagement among pharmacists is critical to improve productivity, prevent turnover, and ensure the quality of community healthcare. Supervising pharmacists weretargeted due to their pivotal role as gatekeepers between organizational leadership and frontline staff in community pharmacies.

**Methods:**

A cross-sectional survey was conducted among supervising pharmacists from major community pharmacy chainsbetween August 2023 and September 2024. Participants completed a web-based questionnaire assessing demographic data, occupational stress, and work engagement. The associations between work engagement and related factors were evaluated using bivariate and multivariate logistic regression analyses with high work engagement defined as the top quartile of UWES-17.

**Results:**

Data from 973 participantswere analyzed. The median UWES-17 score was 2.82. Among the three dimensions, “dedication” scored the highest. The primary factors associated with high work engagement includedperceived “meaningfulness of work”, “job control”, “suitable jobs”, “age group (≥50 years)”, “coworker support”.

**Conclusion:**

This study suggests that the distinct roles and workplace environments of supervising pharmacists are closely linked to their work engagement. Balancing job resources and demands is critical for sustaining engagement to maintain high-quality patient care. Interventions that strengthen self-awareness, peer collaboration, and career development within “Communities of Practice” could reinforce these gains.

## Highlights

• Supervising pharmacists' work engagement was at a level similar to nurse managers in prior studies.
• Dedication was the dimension with the highest score among the three factors of engagement.
• “Meaningfulness of work,” “Job control,” and “Suitable jobs” were the primary predictors of high engagement.
• Age group (≥50 years) and “Coworker support” also significantly contributed to high engagement.
• Fostering “Communities of Practice (CoP)” strengthens dedication and is key to sustainable pharmacy function.

## 1 Background

Japan's rapidly super-aging population is driving a sustained increase in medical demand and creating a pressing need for community-based integrated care systems. In Japan, the community-based integrated care system aims to provide integrated medical care, long-term care, preventive care, housing, and daily living support within each community.^1^

The Ministry of Health, Labour and Welfare's action plan for pharmacists' activities in the community emphasizes a shift from material-centered tasks to interpersonal services, promoting pharmacists' digital transformation, and expanding pharmacists' roles in the community.^2^Internationally, this aligns with the shift towards people-centered pharmaceutical care advocated byaimed by the International Pharmaceutical Federation (FIP).

In this context, supervising pharmacists serve as gatekeepers connecting organizations and the field. Crucially, under Japanese law, they are explicitly mandated to supervise employees, in addition to managing pharmaceuticals and ensuring their proper use.^3^

Furthermore, with the progression of aging in Japan, a significant shortage of professionals and service workers in the medical and welfare sectors has been identified as a major challenge leading to reduced labor productivity and a decline in workers' job satisfaction and motivation. Consequently, efforts to maintain and improve work engagement are recommended to address this issue, emphasizing the need for such measures among pharmacists as well.^4^

Work engagement has been reported to exhibit a crossover phenomenon, where it spreads between individuals in the same environment, such as supervisors and subordinates, such that one person's emotions and attitudes can “transfer” to another person in the same environment.^5^, ^6^, ^7^ Employees construct the meaning of their work by referencing social signals from others in the workplace,^8^ suggesting that supervising pharmacists with high engagement can shape the engagement of their subordinates through daily interactions. Strategically enhancing pharmacists' work engagement is essential not only for productivity and retention but also for sustaining the quality of local healthcare.

Work engagement is defined as a positive and fulfilling work-related state of mind characterized by vigor (high levels of energy and perseverance when faced with difficulties), dedication (experiencing a strong sense of fulfillment, inspiration, pride, and challenge), and absorption (being completely focused on and immersed in one's work.^9^^,^^10^ The positive outcomes of enhancing work engagement include reduced turnover rates; improved health; increased job performance; stronger organizational commitment; and enhanced proactivity, innovation, and creativity.^11^^,^^12^

The Job Demands–Resources (JD-R) model identifies “job demands”, “job resources”, and “personal resources” as key factors influencing work engagement.^11^^,^^13^^,^^14^“Job demands” include job pressure, emotional demands in interpersonal interactions, and mental and physical strain and require sustained effort. “Job resources”, such as autonomy, performance feedback, support from supervisors and coworkers, and career development opportunities, interact with “personal resources”, such as self-efficacy, optimism, resilience, and hope, to enhance work engagement.^11^^,^^13^, ^14^, ^15^

Guided by this framework, for pharmacists to provide high-quality pharmaceutical care in community pharmacies, supervising pharmacists need to pay attention to their working environment and psychological state. Given this role, understanding the work engagement of supervising pharmacists may offer important insights for creating workplaces that enable frontline pharmacists to deliver safe and effective care. Building on this insight, clarifying supervising pharmacists' work engagement, identifying its associated factors, and devising organization-wide measures to foster it constitute pressing priorities for the profession.

Therefore, the purpose of this study was to evaluate the work engagement of supervising pharmacists and, utilizing the Job Demands–Resources model as a theoretical basis, to explore and characterize the work-related factors distinguishing highly engaged supervisors.

## 2 Methods

### 2.1 Study design and setting

This study was conducted as a cross-sectional survey of supervising pharmacists —many of whom concurrently serve as pharmacy managers — at major community pharmacy chains companies between August 2023 and September 2024.

These large insurance-pharmacy chains were chosen because they employ substantial pharmacist workforces, proactively align with national policy, and thus provide a robust sample for capturing sector-wide trends within a defined time-frame. Their organizational scale and operational infrastructure also mean they are likely to shape future community-pharmacy practice, making them a pertinent focus for this study. Specific companies were chosen based on a comprehensive consideration of factors, including the number of pharmacists and the numberof stores.

### 2.2 Participants and eligibility

Supervising pharmacists working in stores more with at least two staff members were included. Specific details regarding the number of pharmacies surveyed, and the exact number of pharmacists and other employees per establishment, cannot be disclosed due to the protection of participant privacy and compliance with ethical guidelines. This represents a limitation regarding data availability.

### 2.3 Survey administration and consent

The survey was conducted using a web-based questionnaire collected anonymously with unique research IDs assigned without correspondence tables. Regarding informed consent, an explanation was provided on the questionnaire form, and the participants were deemed to have consented by responding to the questions. The system was designed not to proceed if consent was not obtained. This procedure constituted implied comprehensive consent. Participants who did not agree were automatically excluded, and no withdrawal requests were received after submission (*n* = 0). Exclusion criteria included withdrawal requests from participants, unidentified gender, and mismatched IDs between questionnaires.

### 2.4 Measures

The survey itemshaveincluded basic attributes of participants, including gender; years of employment; years of professional experience since obtaining a pharmacist's license; duration in the current managerial position; allocation of work hours to dispensing, interpersonal, and managerial work; marital status; presence of children; job title; perceived workload during normal and busy periods; and occupational stress. Allocation of work hours to dispensing, interpersonal, and managerial work was evaluated using a 10-point scale, where participants assigned points to each task, totaling 10 points. Perceived workload during normal and busy periods was assessed using a 5-point scale, ranging from 1 (‘not burdensome at all’) to 5 (‘extremely burdensome’).Occupational stress was measured using the Brief Job Stress Questionnaire provided by the Ministry of Health, Labour, and Welfare.^16^ This questionnaire consists of 19 scales (57 items) categorized into three factors: nine scales related to stress sources, six concerning physical and mental responses to stress, and four reflecting other factors influencing stress responses. Responses to each item were converted into scores on a five-point scale according to a raw score conversion table with higher stress levels corresponding to lower scores (1 point at the lowest) and lower stress levels to higher scores (5 points at the highest).

### 2.5 Work engagement measure

Work engagement was assessed using the original Utrecht Work Engagement Scale (UWES-17) developed by Schaufeli et al., which has been validated for reliability and validity in Japan in the form of the UWES-J.^9^^,^^17^ Each item was rated on a 7-point Likert scale ranging from 0 (“never”) to 6 (“always”). Scores for each subscale (vigor, dedication, absorption) and the total score were calculated as the average of the relevant items; higher values indicated greater work engagement.^9^^,^^17^ In Japan, a simplified version (UWES-9) is widely used because of its simplicity and practical applicability 14).^17^ However, this study adopted the original version, the UWES-17, since the aim was to grasp and analyze the current state of work engagement among supervising pharmacists in depth and to explore the associated factors work engagement using statistical methods in a comprehensive manner.

### 2.6 Statistical analysis

Descriptive statistics were used to determine the proportions of basic attributes, including gender and age. The fundamental trends of the three UWES-17 subscales (vigor, dedication, and absorption) were examined by calculating medians, means, and standard deviations and comparing UWES-17 scores by attributes. Relationships between the UWES-17 and other factors were examined using bivariate and multivariate analyses in JMP® 18.0.1. For bivariate analysis, the UWES-17 scores and basic attributes were set as the dependent and explanatory variables, respectively. For occupational stress analysis, five-point scale scores for the three stress-related categories were aggregated and used as explanatory variables. Each of the 19 scales was scored on a five-point scale (1 = highest stress, 5 = lowest stress), using a MHLW's score conversion table. For multivariate analysis, the responses to each scale were treated as ordinal variables ranging from level 1 to level 5. Pairwise comparisons were conducted between adjacent score levels (e.g., level 3 vs 2, level 5 vs 3) to examine the relative impact of incremental differences in perceived stress or related conditions. In this context, higher levels indicate lower levels of perceived stress or more favorable psychosocial work conditions. These comparisons were designed to detect potential threshold effects within each scale. Spearman's rank correlation coefficient and the Kruskal-Wallis test were used for continuous and nominal scales, respectively, among the dependent variables. Post-hoc pairwise comparisons following the Kruskal-Wallis test were conducted using Dunn's test. For all statistical analyses, the significance level was set to 5 %. The interpretation of correlation coefficients in this study followed psychological research conventions; absolute values of Spearman's rank correlation coefficient were classified into “no correlation” (0), “weak correlation” (0.1–0.3), “moderate correlation” (0.4–0.6), “strong correlation” (0.7–0.9), and “perfect correlation” (1).^18^

### 2.7 Logistic regression modeling

For multivariate analysis, binary logistic regression was conducted, and odds ratios were confirmed for significant factors. First, the quartile ranges of the total UWES-17 scores were calculated with the upper and lower 25 % quartiles set as 1 and 0, respectively, as dependent variables. To identify distinct characteristics of the high-engagement group effectively, high work engagement was defined as the top quartile of UWES-17 scores (comparing the top 25 % vs. the bottom 25 %).

To determine the explanatory variables included in the logistic regression model, bivariate correlations were first examined among all survey variables. When strong correlations were identified, one variable from each highly correlated pair was retained to avoid multicollinearity. In addition, some items in the occupational stress measures were found to conceptually overlap with aspects of work engagement. Therefore, careful discussions were held among the research team to ensure that the selected variables appropriately reflected the objectives of the present study without redundancy. These procedures were directly reflected in the final set of variables included in the logistic regression model. Multicollinearity was considered in setting the explanatory variables for occupational stress, including quantitative and qualitative job overload, perceived physical demands, interpersonal conflict, poor physical environment stress, job control, skill utilization, suitable jobs, meaningfulness of work, supervisor support, coworker support, and support from family and friends. Gender and age, which are found to be related to work engagement in several studies, were added and forcibly entered into the model as control variables. The overall model was tested using the likelihood ratio test at a 5 % significance level. The model fit was evaluated comprehensively based on the local outlier factor, Nagelkerke R^2^, Akaike information criterion (AIC), and Bayesian information criterion (BIC).

### 2.8 Ethics approval

This study has been approved by the Ethics Review Committee of the Nihon Pharmaceutical University (Nihon-Rin 5–7).

## 3 Results

The survey received valid responses from 973 of the 1095 targeted supervising pharmacists (response rate: 88.9 %). Of these, 489 were women, and 484 were men. Table 1 shows the gender-specific age distribution; years of employment; years of professional experience since obtaining a pharmacist's license; duration in the current managerial position; allocation of work hours to dispensing, interpersonal, and managerial work; marital status; presence of children; job titles; and perceived workload during both normal and busy periods.

**Table 1:** Basic data by gender and age group.

	Women						Men						Total					
	20s	30s	40s	50s	60s and above	Total	20s	30s	40s	50s	60s and above	Total	20s	30s	40s	50s	60s and above	Total
Baseline Survey																		
N (%)	51 (5.2)	236 (24.3)	112 (11.5)	70 (7.2)	20 (2.1)	489 (50.3)	70 (7.2)	245 (25.2)	113 (11.6)	45 (4.6)	11 (1.1)	484 (49.7)	121 (12.4)	481 (49.4)	225 (23.1)	115 (11.8)	31 (3.2)	973 (100)
Years of employment (month)	40.5	88.0	150.0	132.0	137.5	89.0	44.0	81.0	156.0	120.0	142.0	76.0	43.0	87.0	151.0	129.0	142.0	88.0
Years of professional experience since obtaining a pharmacist license (month)	46.0	105.0	250.0	344.0	462.0	164.5	45.0	112.0	237.0	341.0	456.0	130.0	46.0	108.5	240.0	343.5	456.0	136.5
Duration in the current managerial position (month)	8.0	28.0	55.5	61.5	87.0	33.0	9.0	45.0	75.0	106.0	120.0	47.0	9.0	36.0	62.0	69.0	96.0	36.5
Effort allocation (Total = 10 points)																		
Dispensing work	3.0	3.0	3.0	3.0	3.5	3.0	3.0	3.0	3.0	3.0	4.0	3.0	3.0	3.0	3.0	3.0	4.0	3.0
Interpersonal work	4.0	4.0	4.0	4.0	4.5	4.0	4.0	4.0	4.0	4.0	4.0	4.0	4.0	4.0	4.0	4.0	4.0	4.0
Managerial work	3.0	3.0	2.0	2.0	2.0	3.0	3.0	3.0	3.0	2.0	2.0	3.0	3.0	3.0	3.0	2.0	2.0	3.0
Marital status (%)	22.9	40.2	57.3	61.8	85.0	47.4	33.3	57.1	78.4	88.4	77.8	61.9	29.1	48.8	67.9	72.1	82.8	54.6
Presence of children (%)	0.0	13.6	49.1	62.9	85.0	30.3	8.6	42.4	71.7	73.3	72.7	47.9	5.0	28.3	60.4	67.0	80.6	39.1
Managerial position (Store Manager or Above) (%)	80.4	61.9	71.4	68.6	80.0	67.7	70.0	66.9	75.2	75.6	63.6	70	74.4	64.4	73.3	71.3	74.2	68.9
Perceived workload during normal periods (5-point scale)	3.0	3.0	3.0	4.0	3.5	3.0	3.0	3.0	3.0	3.0	3.0	3.0	3.0	3.0	3.0	3.0	3.0	3.0
Perceived workload during busy periods (5-point scale)	4.0	4.0	4.0	4.0	4.0	4.0	4.0	4.0	4.0	3.0	4.0	4.0	4.0	4.0	4.0	4.0	4.0	4.0

This table presents baseline survey results, including years of employment, managerial positions, and perceived workload, categorized by gender and agegroup. The “Total” column indicates the sum of participants (N) for women, men, and the overall sample, while the other values represent the mean scores for each group. No notable differences were observed in the overall male-to-female ratio.

Occupational stress analysis revealed that men reported high stress levels in “quantitative job overload” and “fatigue”, while women reported high stress levels in “quantitative job overload and qualitative job overload” with over 25 % of respondents in these categories classified as having high stress (Table 2; Additional files1). Regarding workplace support, the proportion of high stress ratings was relatively low. The proportions of high stress related to support from supervisors were 4.1 % for men and 1.2 % for women. The proportions of support from colleagues were 7.0 % for men and 1.8 % for women. The proportions of support from family and friends were 7.9 % for men and 3.3 % for women.

**Table 2:** Gender-specific results of the brief job stress questionnaire (BJSQ).

	Women						Men					
	1	2	3	4	5	Proportion of High-Stress Levels	1	2	3	4	5	Proportion of High-Stress Levels
A Job stressors												
Quantitative job overload	127	119	209	32	2	**26.****0 %**	136	125	160	46	17	**28.****1 %**
Qualitative job overload	234	179	58	18		**47.****9 %**	91	192	160	34	7	18.8 %
Physical demands	84	197	175	33		17.2 %	48	162	200	74		9.9 %
Interpersonal conflict	8	56	224	159	42	1.6 %	25	65	218	134	42	5.2 %
Poor physical environment	29	82	211		167	5.9 %	39	68	207	170		8.1 %
Job control	12	52	259	134	32	2.5 %	42	104	203	111	24	8.7 %
Skill utilization	8	38	263	180		1.6 %	10	80	247	147		2.1 %
Suitable jobs	19	107	296		67	3.9 %	16	132	281		55	3.3 %
Meaningfulness of work	7	84	304		94	1.4 %	35	143	236		70	7.2 %
B Stress responses												
Vigor	63	57	190	152	27	12.9 %	76	49	212	127	20	15.7 %
Anger-irritability	28	126	199	74	62	5.7 %	50	146	165	50	73	10.3 %
Fatigue	66	198	173	42	10	13.5 %	126	156	150	22	30	**26.****0 %**
Anxiety	33	149	208	36	63	6.7 %	28	136	210	49	61	5.8 %
Depression	39	163	174	57	56	8.0 %	57	132	172	67	56	11.8 %
Physical stress reaction	78	166	145	73	27	16.0 %	107	105	153	93	26	22.1 %
C Social support												
Supervisor support	6	22	114	240	107	1.2 %	20	66	110	192	96	4.1 %
Coworker support	9	64	181	150	85	1.8 %	34	89	199	108	54	7.0 %
Support from family and friends	16	28	98	134	213	3.3 %	38	52	104	122	168	7.9 %
Job satisfaction	10	51	313	80	35	2.0 %	22	74	296	68	24	4.5 %

This table presents the gender-specific results of the Brief Job Stress Questionnaire, focusing on job stressors, stress responses, and social support.

Scores for each BJSQ scale were converted to a five-point scale (levels 1–5) using the Ministry of Health, Labour and Welfare score conversion table, where level 1 represents the highest stress level and level 5 represents the lowest stress level. “High stress (%)” indicates the proportion of respondents classified in level 1 for each scale. Among men, more than 25 % were classified as experiencing high stress related to Quantitative Job Overload and Fatigue. Among women, over 25 % experienced high stress in both Quantitative and Qualitative Job Overload with nearly half classified as experiencing high stress in Qualitative Job Overload. Additionally, lower levels of supervisor and coworker support were associated with higher stress levels, particularly in men (4.1 % and 7.0 %, respectively) compared to women (1.2 % and 1.8 %, respectively).

The results of the UWES-17 are presented in Table 3. The median total score on the UWES-17 was 2.82 (mean ± SD: 2.80 ± 0.98). Men had a median score of 2.74 (mean ± standard deviation [SD]: 2.68 ± 1.00), and women had a median score of 2.94 (mean ± SD: 2.92 ± 0.93). Among the three factors (vigor, dedication, and absorption), “dedication” was highest, followed by “absorption” and “vigor”. Regarding dedication, the overall median was 3.00 (mean ± SD: 3.00 ± 1.14). For women, the median was 3.20 (mean ± SD: 3.16 ± 1.09), and for men, the median was 2.80 (mean ± SD: 2.84 ± 1.17). Regarding absorption, the overall median was 2.83 (mean ± SD: 2.78 ± 1.05). For women, the median was 2.83 (mean ± SD: 2.90 ± 1.01), and for men, the median was 2.67 (mean ± SD: 2.65 ± 1.09). Regarding vigor, the overall median was 2.67 (mean ± SD: 2.66 ± 1.03). For women, the median was 2.67 (mean ± SD: 2.74 ± 1.00), and for men, the median was 2.50 (mean ± SD: 2.58 ± 1.06).

**Table 3:** Distribution of UWES-17 scores by gender and age group.

		Women							Men							Total					
		20s	30s	40s	50s	60s and above	Total		20s	30s	40s	50s	60s and above	Total		20s	30s	40s	50s	60s and above	Total
N		51	236	112	70	20	489		70	245	113	45	11	484		121	481	225	115	31	973
Work engagement score per item																					
Vigor	Median	2.8	2.7	2.7	3.1	3.3	2.7		2.3	2.7	2.5	2.8	2.7	2.5		2.5	2.7	2.7	3.0	3.2	2.7
	Mean	2.7	2.6	2.8	3.1	3.3	2.7		2.5	2.5	2.5	3.0	2.9	2.6		2.6	2.6	2.6	3.1	3.2	2.7
	SD	0.9	1.0	0.9	1.1	0.8	1.0		1.2	1.0	1.0	1.1	0.9	1.1		1.1	1.0	1.0	1.1	0.8	1.0
Dedication	Median	3.2	3.0	3.2	3.5	3.7	3.2		2.8	2.8	2.8	3.2	3.6	2.8		3.0	3.0	3.0	3.2	3.6	3.0
	Mean	3.1	3.0	3.2	3.6	3.7	3.2		2.8	2.8	2.7	3.3	3.3	2.8		2.9	2.9	3.0	3.5	3.6	3.0
	SD	0.9	1.1	1.0	3.6	0.9	1.1		1.3	1.1	1.1	1.0	0.9	1.2		1.2	1.1	1.1	1.1	0.9	1.1
Absorption	Median	2.8	2.8	2.9	3.6	3.3	2.8		2.7	2.7	2.7	3.0	2.7	2.7		2.8	2.7	2.8	3.2	3.2	2.8
	Mean	2.9	2.8	2.9	3.6	3.2	2.9		2.7	2.6	2.7	3.0	2.9	2.7		2.8	2.7	2.8	3.2	3.1	2.8
	SD	0.9	1.0	1.0	3.6	1.0	1.0		1.2	1.1	1.0	1.1	1.0	1.1		1.1	1.1	1.0	1.1	1.0	1.1
Total	Median	2.9	2.8	2.9	3.6	3.4	2.9		2.6	2.7	2.6	2.9	3.0	2.7		2.8	2.8	2.8	3.1	3.3	2.8
	Mean	2.9	2.8	3.0	3.6	3.4	2.9		2.6	2.6	2.6	3.1	3.0	2.7		2.7	2.7	2.8	3.2	3.3	2.8
	SD	0.8	1.0	0.9	3.6	0.8	0.9		1.1	1.0	1.0	1.0	0.8	1.0		1.0	1.0	0.9	1.0	0.8	1.0
Total work engagement score																					
Vigor	Median	17.0	16.0	16.0	18.5	19.5	16.0		14.0	16.0	15.0	17.0	16.0	15.0		15.0	16.0	16.0	18.0	19.0	16.0
	Mean	16.2	15.4	16.7	18.7	19.9	16.4		14.7	15.3	15.1	18.2	17.6	15.5		15.3	15.4	15.9	18.5	19.1	16.0
	SD	5.6	6.0	5.6	6.3	4.8	6.0		6.9	6.2	6.2	6.5	5.1	6.4		6.4	6.1	6.0	6.3	4.9	6.2
Dedication	Median	16.0	15.0	16.0	17.5	18.5	16.0		14.0	14.0	14.0	16.0	18.0	14.0		15.0	15.0	15.0	16.0	18.0	15.0
	Mean	15.3	14.9	16.1	17.9	18.5	15.8		13.8	14.1	13.6	16.4	16.7	14.2		14.4	14.5	14.8	17.3	17.9	15.0
	SD	4.5	5.6	5.2	5.4	4.7	5.4		6.6	5.7	5.7	5.2	4.4	5.8		5.8	5.7	5.6	5.3	4.6	5.7
Absorption	Median	17.0	16.5	17.5	19.5	20.0	17.0		16.0	16.0	16.0	18.0	16.0	16.0		17.0	16.0	17.0	19.0	19.0	17.0
	Mean	17.6	16.6	17.4	19.5	19.3	17.4		15.9	15.5	15.9	18.2	17.4	15.9		16.6	16.0	16.6	19.0	18.6	16.7
	SD	5.1	6.1	5.8	6.3	6.1	6.0		7.3	6.5	6.1	6.3	5.8	6.5		6.5	6.3	6.0	6.3	6.0	6.3
Total	Median	50.0	47.0	50.0	55.0	57.5	50.0		44.0	46.0	44.0	50.0	51.0	46.5		47.0	47.0	48.0	52.0	56.0	48.0
	Mean	49.0	47.0	50.2	56.0	57.7	49.6		44.4	44.8	44.6	52.8	51.7	45.6		46.4	45.9	47.4	54.7	55.5	47.6
	SD	13.6	16.2	14.9	16.3	13.8	15.9		18.7	16.7	16.5	16.4	14.2	17.0		16.8	16.5	16.0	16.3	14.0	16.6

This table presents the distribution of UWES-17 scores by gender and age group, focusing on work engagement scores per item and total scores. The data include median, mean, and standard deviation (SD) values for each category. The median total work engagement score was 2.82 (mean ± SD: 2.80 ± 0.98). For men, the median score was 2.74 (mean ± SD: 2.68 ± 1.00), while women showed a higher median score of 2.94 (mean ± SD: 2.92 ± 0.93). Among the three components of work engagement, dedication had the highest scores, followed by absorption and vigor.

The “Total” column indicates the sum of participants (N) for women, men, and the overall sample, while the other values represent the median and mean scores for each group. The “Total” row shows the mean scores of the items.

The relationships between the UWES-17 scores and the basic attributes are shown in Additional files 2–5. Analysis of the UWES-17 scores and occupational stress revealed significant correlations across all items. Analysis by age group showed that participants in their 50s and those aged 60 and above had significantly higher work engagement than those in their 20s–40s. An analysis of perceived workload during normal and busy periods revealed significant differences in work engagement between groups reporting a “very high workload” (Group 5) and other groups (Groups 1–3). Participants with spouses showed significantly higher work engagement overall, and those with children exhibited significantly higher scores across all subscales of the UWES-17.

The results of the binary logistic regression analysis are presented in Table 4. For the logistic regression analysis, the quartile distribution of the total UWES-17 scores was calculated to define the binary outcome variable. The median score was 48.0, with the first quartile (Q1) at 37.0 and the third quartile (Q3) at 58.0, indicating an interquartile range of 21.0. Participants whose total scores were above the third quartile (Q3 > 58.0) were classified into the high engagement group (*n* = 236), while those with scores below the first quartile (Q1 < 37.0) were classified into the low engagement group (*n* = 258). These groups were assigned values of 1 and 0, respectively, and used as the dependent variable in the logistic regression model. “Meaningfulness of work”, “job control”, “suitable jobs”, and “age group (≥50 years)” were identified as significant factors that increased the likelihood of being in the upper quartile of UWES-17 scores. For “meaningfulness of work”, the odds were 15.4 times higher (*p* < 0.0001) when the score increased from 2 to 3 and 15.6 times higher (p < 0.0001) when it increased from 3 to 5. For “suitable jobs”, an increase from 2 to 3 raised the odds by 4.1 times (*p* = 0.0003); for “job control”, the same increase raised the odds by 3.5 times (*p* = 0.0037). Participants aged 50 years and above had 3.7-times-higher odds (*p* = 0.0031) compared with those under 50 years. Supplementary factors included “coworker support”, “poor physical environment stress”, and “quantitative job overload”. The model fit indicators were R^2^ (0.509), AIC (444.0), and BIC (635.4).

**Table 4:** Predictors influencing high work engagement: A logistic regression analysis.

Variable	Comparison (Level)	Estimate (β)	Std. Error	χ^2^	*p*-value	Odds Ratio (OR)	95 % CI (Lower)	95 % CI (Upper)
Meaningfulness of work	3 vs 2	2.73	0.45	36.81	<0.0001	15.4	6.67	39.5
	5 vs 3	2.75	0.67	16.63	<0.0001	15.6	4.56	65.48
Suitable jobs	3 vs 2	1.42	0.39	13.35	0.0003	4.14	1.96	9.09
Age group	<50 vs ≥50	−0.66	0.22	8.72	0.0031	0.52	0.33	0.79
Job control	3 vs 2	1.26	0.43	8.41	0.0037	3.52	1.52	8.42
Coworker support	5 vs 4	1.43	0.54	7.07	0.0078	4.17	1.49	12.35
Poor physical environment stress	4 vs 3	−1.06	0.48	4.79	0.0286	0.35	0.13	0.89
Quantitative job overload	5 vs 4	3.33	1.65	4.07	0.0437	27.82	1.18	1056.4

This table presents the results of the binary logistic regression analysis, highlighting the statistically significant factors influencing high work engagement. The predictors, excluding the age group variable, were treated as ordinal variables with five levels. The overall model fit indices (pseudo R^2^ = 0.509, AIC = 444, BIC = 635.4) and the model's global significance (p < 0.0001) indicate that the model adequately explains the dependent variable. The intercept (β = −37.35, *p* = 0.9876) and parameters excluded from the table were not statistically significant. The instability of the intercept does not affect the interpretation of the explanatory variables. While the predictors “Coworker Support”, “Poor physical environment stress”, and “Quantitative job overload” were not significant overall, specific pairwise comparisons within these variables demonstrated statistically significant effects on the outcome. *p* < 0.05 was considered statistically significant. In this table, ‘Comparison (Level)’ indicates the specific comparison between levels of each predictor variable used in the logistic regression analysis. For example, ‘3 vs 2’ refers to level 3 being compared to level 2 as the reference category, and ‘5 vs 3’ refers to level 5 being compared to level 3.

## 4 Discussion

This study clarified the actual state of work engagement among supervising pharmacists in major insurance pharmacy companies and identified the associated factors.

First, the work engagement of supervising pharmacists was examined based on previous studies conducted in other industries. The median UWES-17 score of supervising pharmacists was 2.82. According to Shimazu and other previous studies, the UWES-9 score for general Japanese workers is estimated to be approximately 2.8 to 2.9.^5^^,^^19^^,^^20^

In healthcare professions, general nurses have reported scores ranging from 2.2 to 2.7.^21^, ^22^, ^23^, ^24^

A study across 16 facilities reported that general nurses had a median score of 2.4, while nurse managers had a median score of 3.0, suggesting that higher positions tended to have higher scores.^24^ Although direct comparisons were not possible, the work engagement of supervising pharmacists was similar to that of general Japanese workers and nurse managers.

A cross-national study using UWES-9 data from 16 countries, including Japan, reported that Japanese workers scored significantly lower in work engagement than workers in the other 15 countries.^25^ Another study further showed that Japanese UWES-9 scores were lower than those in other countries, that the Japanese version of UWES-9 exhibits reduced measurement precision at low levels of engagement, and that Japanese individuals may have a cultural tendency to report positive emotions more modestly.^20^ Therefore, when interpreting work engagement scores among Japanese workers, these cultural factors and the measurement properties of the scale should be taken into consideration.

In this study, over 25 % of the participants were identified as experiencing high stress due to “quantitative job overload”.Amid chronic staffing shortages caused by pharmacy expansion and regional disparities, supervising pharmacists must juggle players and managerial roles and handle a wide range of pharmacy operations, which cause high job demands.

Strengthening systems that utilize job resources and enhance support for supervising pharmacists is essential to reduce stress reactions and improve work engagement (Table 2, Table 4).

The binary logistic regression analysis in this study identified seven factors that significantly influenced the work engagement of supervising pharmacists: “meaningfulness of work,” “suitable jobs,” “job control,” “coworker support,” “poor physical environment stress,” “quantitative job overload,” and “age group” (Table 4). These factors highlight the challenges in the roles and work environments unique to supervising pharmacists in Japan, as framed by the Job Demands-Resources (JD-R) model, which encompasses both Job Resources and Job Demands.

First, factors classified as Job Resources include “suitable jobs,” “job control,” “coworker support,” and “meaningfulness of work.”

Regarding “suitable jobs” and “job control,” the issue lies in the fact that restrictions on the supervising pharmacist's unique decision-making and adjustment roles due to management policies are reportedly undermining the autonomy of pharmaceutical operations and hindering the utilization of individual capabilities.^26^

“Coworker support” is a critical Job Resource, but the chronic staffing shortages in pharmacies limit opportunities for meetings involving all employees and informal communication, creating an environment where adequate support from colleagues is difficult to obtain.

Meanwhile, “meaningfulness of work” (Job satisfaction) was an important factor observed in the high engagement group. Engaging in meaningful work is thought to be indispensable for enhancing work engagement by boosting Personal Resources such as “self-efficacy” and “optimism” within the JD-R model. To enhance “meaningfulness of work,” it is necessary to deliberately provide “opportunities for decision-making accompanied by a sense of contribution” and “opportunities for collaborative knowledge creation,” where supervising pharmacists can leverage their expertise and feel they are contributing to the entire pharmacy or regional healthcare. Specifically, forming a “Community of Practice” that combines “coworker support,” the importance of which was demonstrated in this study, within the organization provides an effective foundation for promoting knowledge sharing and mutual understanding, thereby fostering an organizational climate where individual pharmacists can redefine the purpose of their roles.^27^

Next, factors affecting higher work engagement included “quantitative job overload” and “poor physical environment stress.”

“Quantitative job overload” is classified as a Job Demand in the JD-R model. Supervising pharmacists must balance their roles as playing managers with a wide range of pharmacy operations, which creates a high job demand, with over 25 % of participants reporting high stress due to this. While a high workload can be an opportunity for growth, it was also suggested to contribute to stress.

Similarly, prior research demonstrated that stress in community pharmacists significantly impacts patient care quality and safety perceptions.^28^

Regarding “poor physical environment stress,” factors such as a lack of well-equipped back-office space and long working hours in confined areas in typical Japanese pharmacies are likely contributors.

Furthermore, “age group,” which showed a significant influence outside the JD-R model framework, suggests that the accumulation of Personal Resources accompanying years of experience and stability of roles in the later stages of a career may contribute to engagement, supporting the need for human resource strategies tailored to the career stages of pharmacists.

While efforts have been made in recent years to reduce the burden of product-related tasks through the delegation of dispensing work to clerical staff and the introduction of IT tools,^26^ initiatives for reducing the mental burden on supervising pharmacists and developing career education programs are still in their early stages.

To enhance work engagement, individualized measures are essential, such as human resource strategies tailored to the career aspirations of pharmacists, and the cultivation of “learning communities,” including “coworker support” critical in their success and those of staff^26^^,^^29^^,^^30^ and likewise helps to provide an avenue for greater, or more effective leadership that promotes continuity among all personnel.^31^

As a future challenge, it is necessary to verify the relationship between work engagement and proactive individual behaviors, such as Job Crafting, and their impact on organizational commitment among supervising pharmacists.

### 4.1 Limitations

Our sample was drawn from four large insurance-pharmacy chains, excluding the small- and medium-sized pharmacies that account for most outlets nationwide; the findings should therefore be extrapolated only with caution. Additionally, a straightforward comparison with other countries is difficult due to the unique characteristics of the Japanese healthcare system and the specific functions of pharmacists in Japan.

## 5 Conclusions

In order to mitigate the impact of staffing shortages and high job demands, future human resource development should focus not only on cultivating “learning individuals” who excel in specialized skills but also on fostering “learning communities” or “Communities of Practice”that enable teamwork.^27^

Foundational to this is self- and mutual understanding, which fosters organizational climates where individuals find purpose in their work. Given that “coworker support” and “meaningfulness of work” were identified as key factors in this study, our findings suggest that by sharing knowledge and supporting each other within these communities, pharmacists may be able to enhance their “dedication”—the highest scoring dimension in this study—through the intrinsic motivation provided by a sense of meaningfulness, and strengthen their resilience against job demands.

## CRediT authorship contribution statement

**Yukari Ito:** Conceptualization, Writing – original draft. **Hiroko Suzuki:** Writing – review & editing. **Tetsuro Yumoto:** Methodology, Writing – review & editing. **Sachiko Ohta:** Data curation, Formal analysis, Investigation, Writing – review & editing. **Tomoo Hosoe:** Conceptualization, Project administration, Supervision, Writing – review & editing.

## Funding

This research did not receive any specific grant from funding agencies in the public, commercial, or not-for-profit sectors.

## Declaration of competing interest

This research did not receive any specific grant from funding agencies in the public, commercial, or not-for-profit sectors.

All authors declare that they have **no competing financial interests or personal relationships** that could have appeared to influence the work reported in this paper.

## Data Availability

Research data will not be made publicly available to protect the participants' privacy and to comply with ethical guidelines. Moreover, informed consent obtained from the participants did not include permission for data sharing.

## Appendix A Supplementary data

Supplementary material Image 1
