# Supplementary material for: Exploring work engagement and its associated factors among supervising pharmacists in Japan
Source: Explor Res Clin Soc Pharm. 2025 Dec 5;21:100691. doi: 10.1016/j.rcsop.2025.100691 (PMC12757613; doi:10.1016/j.rcsop.2025.100691)
Supplement: Supplementary file 1 — Supplementary material [file mmc1.docx]

**Additional files**

**RESULT**

**Additional files 1 Basic data by gender and age group（BJSQ）**

This table summarizes the findings of the Brief Job Stress Questionnaire, which assesses job stressors, stress responses, and social support. All scores are reported as medians with higher scores indicating better conditions or lower levels of stress. “Total” values represent mean scores of the relevant BJSQ domains after conversion to a 5-point scale. Therefore, the totals may not exactly match the simple sums or averages of the subgroup values due to score conversion and rounding.

**Additional files 2 Correlations between UWES-17 and basic attributes (Continuous variables)**

This table presents the correlations between UWES-17 scores and basic attributes (continuous variables). Correlations are shown for vigor, dedication, absorption, and total scores. The table also includes results from the Brief Job Stress Questionnaire analyzed by subcategories based on a five-point Likert scale. A background color was applied when the correlation coefficient exceeded an absolute value of 0.2 and *p* < 0.05. In the analysis of the UWES-17 scores and occupational stress, significant correlations were observed for all items.*p* < .05 was considered statistically significant.

**Additional files 3-5 Relationships between UWES-17 and basic attributes (Categorical and ordinal variables)**

This table presents the relationships between UWES-17 scores and categorical or ordinal variables, focusing on gender, age group, perceived workload, and demographic/job-related factors.

**Additional files 3 Gender and age group**
Scores for vigor, dedication, and absorption were significantly higher in individuals aged 50 and above compared to those under 50 (*p* < .05).

**Additional files 4 Perceived workload during normal and busy periods**
All values are presented as medians. In the analysis based on the perceived burden of working hours, significant differences in work engagement were observed between the group that reported "very high burden" (Group 5) and the other groups (Groups 1–3) during regular and busy periods.

**Additional files 5 Demographic and job-related factors**
Participants with spouses had significantly higher total work engagement scores, whereas those with children scored significantly higher across all UWES-17 subscales (vigor, dedication, and absorption). *p* < .05 was considered statistically significant.

**Additional files 1 Basic data by gender and age group（BJSQ）**


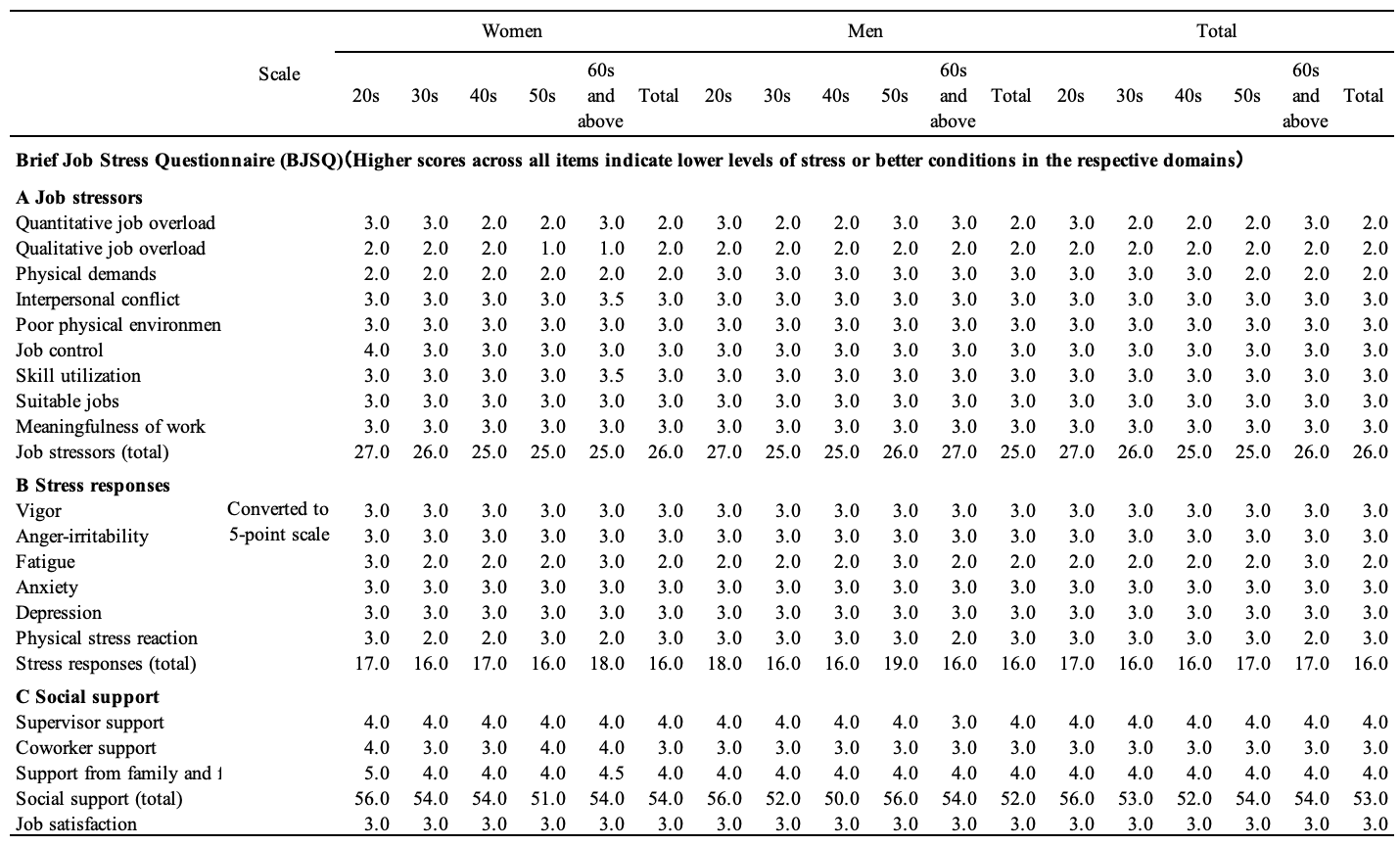


**Additional files 2 Correlations between UWES-17 and basic attributes (Continuous variables)**

**
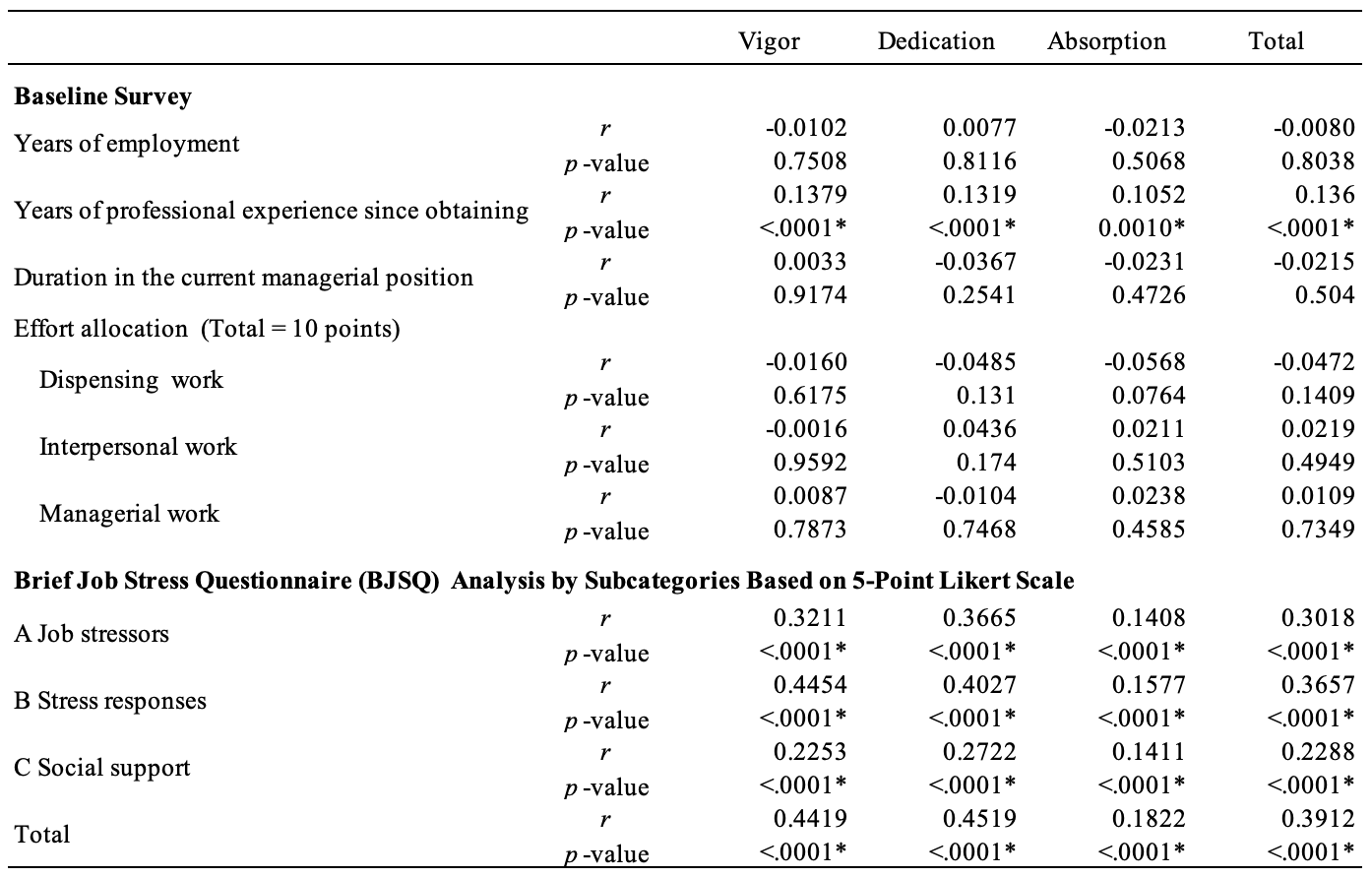
**

**Additional files 3-5 Relationships between UWES-17 and basic attributes (Categorical and ordinal variables)**

**Additional files 3 Gender and age group**

**
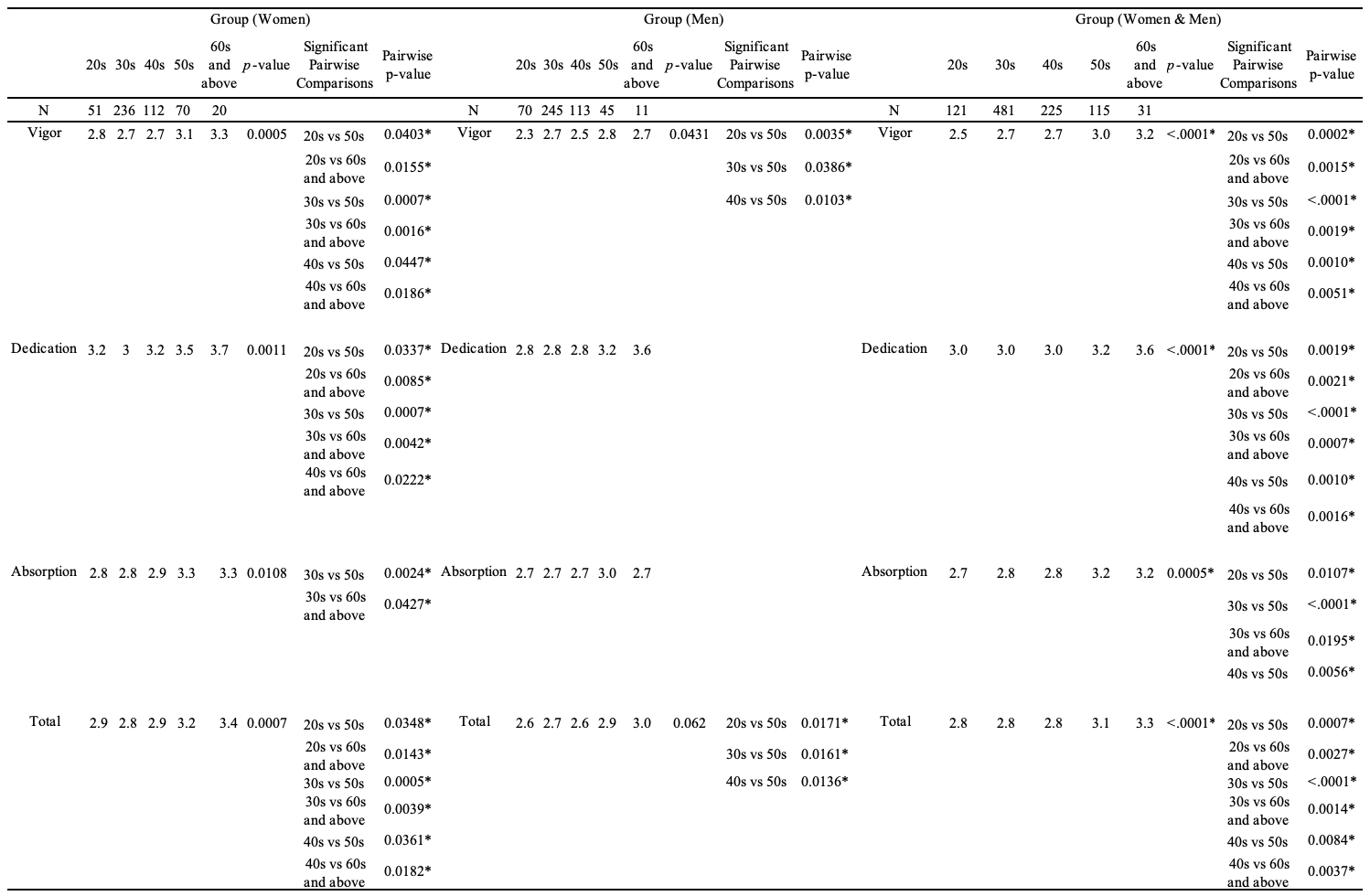
**

**Additional files 4 Perceived workload during normal and busy periods**

**
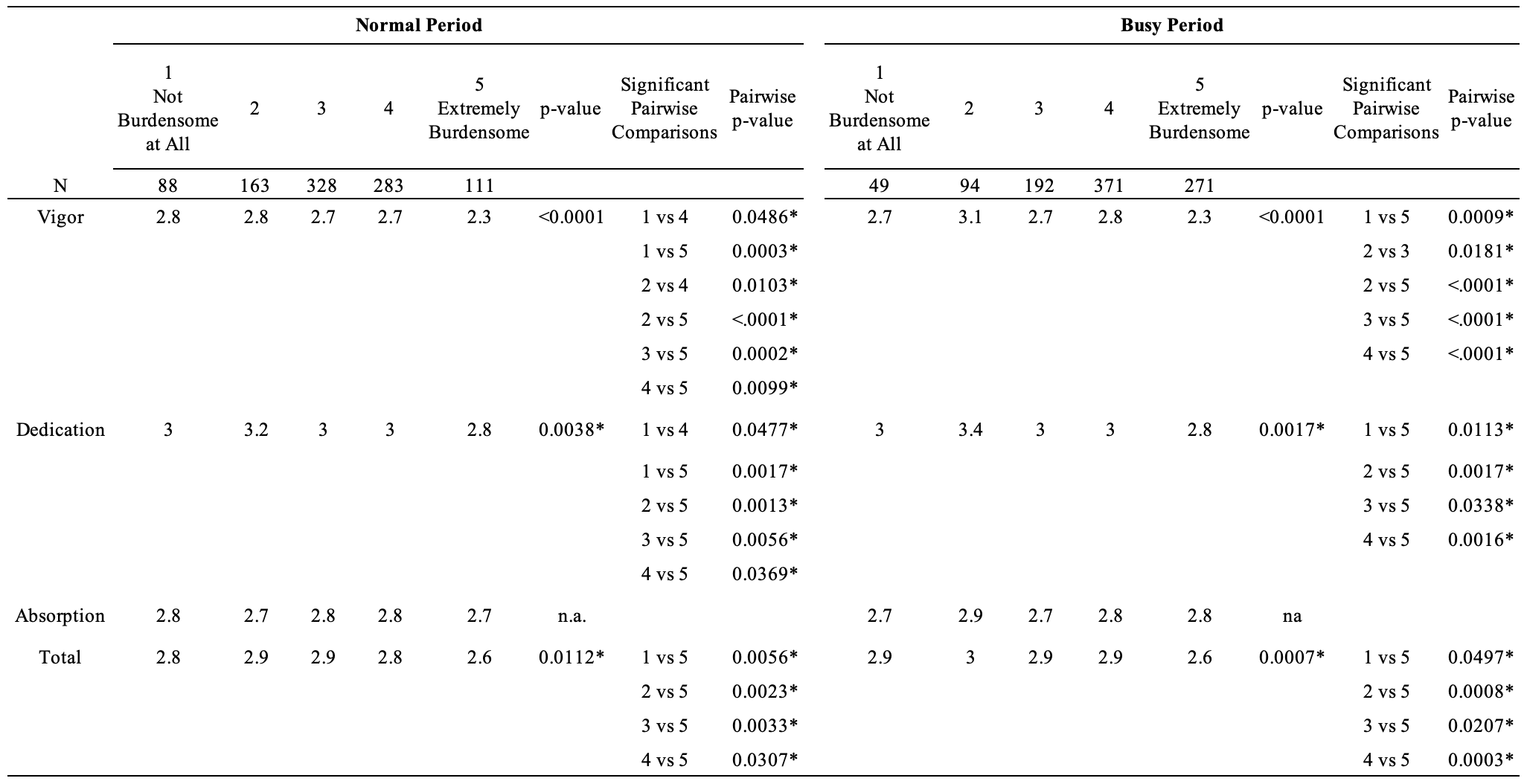
**

**Additional files 5 Demographic and job-related factors**
**
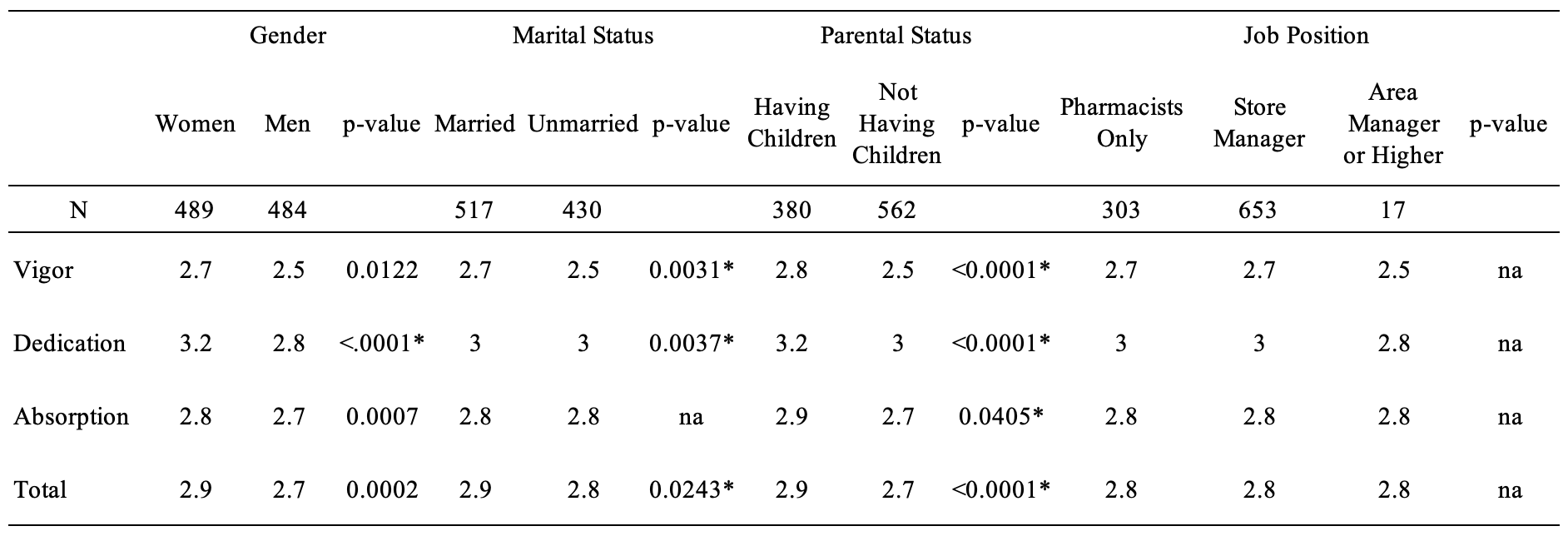
**
